# Inter- vs. Intra-Molecular Hydrogen Bond in Complexes of Nitrophthalic Acids with Pyridine

**DOI:** 10.3390/ijms24065248

**Published:** 2023-03-09

**Authors:** Kinga Jóźwiak, Aneta Jezierska, Jarosław J. Panek, Andrzej Kochel, Aleksander Filarowski

**Affiliations:** Faculty of Chemistry, University of Wrocław, 14 F. Joliot-Curie Str., 50-383 Wrocław, Poland

**Keywords:** phthalic acid, proton dynamics, hydrogen bond, steric repulsion, X-ray, IR, Raman, CPMD, DFT

## Abstract

This study covers the analysis of isomeric forms of nitrophthalic acids with pyridine. This work dwells on the complementary experimental (X-ray, IR and Raman) and theoretical (Car-Parrinello Molecular Dynamics (CPMD) and Density Functional Theory (DFT)) studies of the obtained complexes. The conducted studies showed that steric repulsion between the nitro group in ortho-position and the carboxyl group causes significant isomeric changes. Modeling of the nitrophthalic acid—pyridine complex yielded a short strong intramolecular hydrogen bond (SSHB). The transition energy from the isomeric form with an intermolecular hydrogen bond to the isomeric form with an intramolecular hydrogen bond was estimated.

## 1. Introduction

Hydrogen bonding is the most significant non-covalent interaction responsible for the structure and properties of supramolecular assemblies, from liquid water to protein complexes or DNA. The significant strength of the intramolecular hydrogen bond increases under the influence of various factors. For example, the steric effect reduces the length between the proton-donor and proton-acceptor atoms, which means strengthening the hydrogen bond [1]. Comprehensive studies of the steric effect on the strength of hydrogen bonds in ortho-hydroxy Schiff bases, ortho-hydroxy acetophenones, salicylamides and proton sponges were presented in refs [1,2,3,4,5,6,7,8,9,10,11,12,13,14,15,16,17]. In papers by Pozharskii et al. [2,3] two types of the steric effect on the NHN hydrogen bond were investigated. The shortest NHN hydrogen bond in proton sponges was obtained by means of the combination of “Buttressing” and “Clothespin” effects in the complex of the tetrafluoroborate with 2,4,5,7-tetramethyl-1,8-bis-(dimethylamino)naphthalene [4]. Hansen et al. [5] discovered a unique phenomenon of the deutero-replacement in the methyl group (CH_3_, CH_2_D, CHD_2_ and CD_3_) in some o-hydroxyacylaromatic compounds leading to a low-field shift of the OH resonance. This phenomenon was explained by the different steric impacts of the methyl group on the intramolecular hydrogen bond in the o-hydroxyacylaromatic compounds [5,6,7]. The studies by Filarowski et al. [8,9,10,11] are devoted to the influence of steric repulsion on quasi-aromatic hydrogen bonding in ortho-hydroxy aryl ketoimines and acetophenones. The abovementioned compounds are rather stable in terms of conformational changes. The steric substitution leading to various types of complexes of isopropylamine with the alicyclic ethers was studied [12]. In the paper by Liu et al. [13] the impact of two motifs on the conformational states of lactams—the steric effect and the strength of hydrogen bond—was shown. Of interest are the studies of hydrogen bonding in sterically hindered alcohols [14] and the prevalence of bifurcated hydrogen bonds over the steric effect in self-assembling processes in Schiff bases [15,16]. However, a strong steric effect in the salicylamides evokes a disruption of intramolecular hydrogen bonding due to a rather weak coupling between the hydroxyl group and amide fragment caused by the competing mesomeric effects (−M and +M) [16,17,18,19,20]. Besides, extensive studies of the influence of hydrogen bonding on the isomeric state of acylaminopyridines were performed by Ośmiałowski et al. [21,22,23,24,25,26]. In addition, phthalic acids and lactams are interesting in terms of conformational changes [27,28,29,30,31,32,33,34]. These compounds contain two carboxyl groups not coupled together and, therefore, the steric repulsion can induce significant conformational changes. Such changes are responsible for diverse processes also in the biomedical context. In particular, the steric effect can cause serious diseases such as Alzheimer's disease due to inadequate protein folding. The altered protein tau is characterized by the ability to conformational changes due to the presence of a steric effect causing abnormalities [35,36]. It is worth mentioning the work by Bickelhaupt et al. [37], where a significant role of the hydrogen bond and steric repulsion in DNA replication was elucidated. Transition-metal catalyzed cross-coupling reactions were studied in ref. [38] and the obtained results indicated a steric attraction between the bulk substituents and the catalyst stabilizes the reaction barrier of the C-X bond activation. Sola et al. [39] analyzed the steric impact on the isomerization of diazacyclobutadienes and pyrazoles. It was revealed that the steric effect (Pauli repulsions) does not always play a decisive role in the stabilization of certain types of isomers, thus, the analysis of various non-covalent interactions is very important.

**Figure 1:**
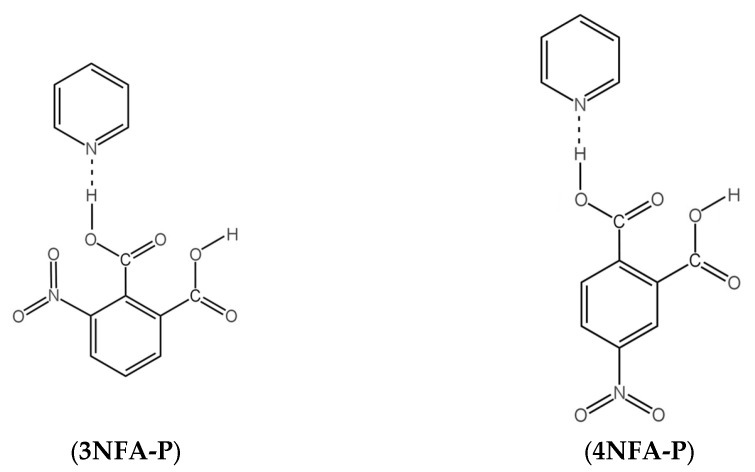
Chemical structures of 3-nitrophthalic (**3NFA-P**) and 4-nitrophthalic (**4NFA-P**) acid complexes with pyridine.

This work is concerned with the studies of the phthalic acids’ complexes (Figure 1). The studies cover the issues of equilibrium between intramolecular hydrogen bonds and intermolecular hydrogen bonds using CPMD and DFT calculations in the solid state (for the structures measured by the X-ray method) and in the gas phase, respectively.

## 2. Results and Discussion

### 2.1. Strength of Hydrogen Bonds and Proton Dynamics in the Studied Complexes

**Figure 2:**
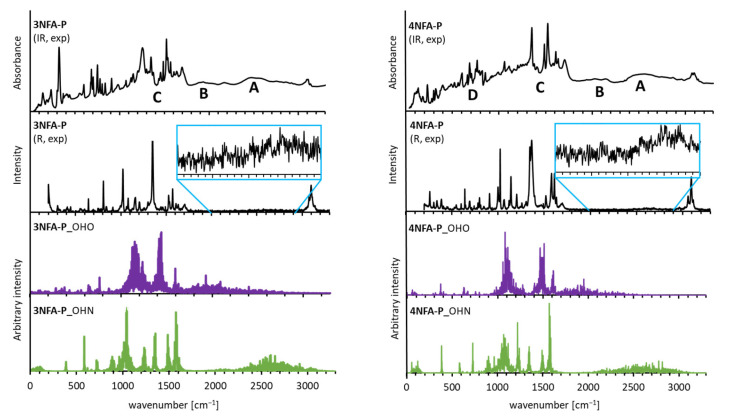
Experimental IR (IR, exp), Raman (R, exp) and atomic velocity power spectra (**3NFA-P**_OHO/OHN and **4NFA-P**_OHO/OHN) for the hydrogen-bonded protons of **3NFA-P** and **4NFA-P** complexes are presented on **left** and **right** panels, respectively. **A**, **B**, **C** and **D** denote the bands related to the strong hydrogen bond, discussed in the text.

**Figure A1:**
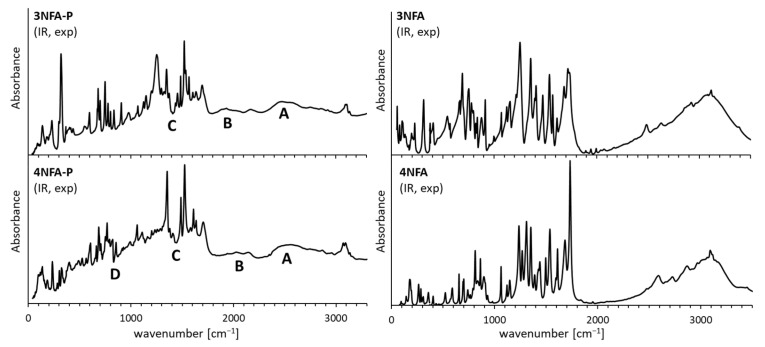
Experimental IR spectra of **3NFA-P**, **4NFA-P** complexes (**left** panels) and **3NFA**, **4-NFA** (**right** panels) compounds.

**Figure A2:**
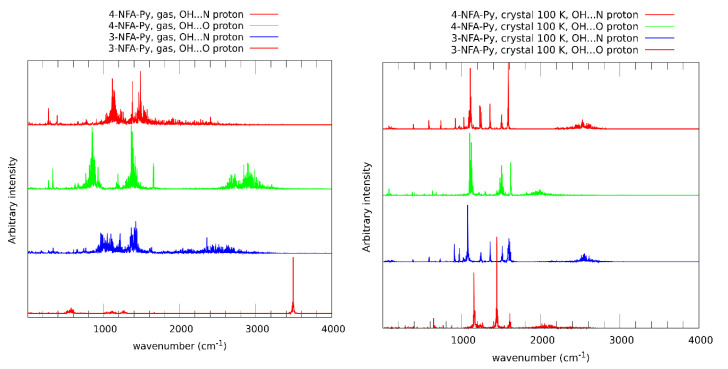
Calculated power spectra of atomic velocity—results of CPMD runs for the **3NFA-P** and **4NFA-P** complexes in the gas phase and in the solid state (T = 100 K). CPMD power spectra are presented only for the bridged protons vibrational modes.

This part of the work includes the analysis of the hydrogen bond strength in the studied complexes based on spectral data. To complete the analysis, both IR and Raman measurements and CPMD simulations have been fulfilled. Figure 2 shows the spectra with the broad bands observed. These broad bands expose features of the so-called Zundel continuum absorption [40], thus indicating the formation of very strong low-barrier hydrogen bonds (LBHB) or the so-called Speackmann–Hadži hydrogen bond [41]. According to the literature [42], these bands are marked as **A**, **B**, **C** and **D** for the **4NFA-P** complex and **A**, **B** and **C** for the **3NFA-P** complex (Figure 2 and Figure A1). Notably, the ν(XH) bands in the spectrum of the **4NFA-P** complex are more red-shifted than the bands in the spectrum of the **3NFA-P** complex, indicating that the hydrogen bonds in the **4NFA-P** complex are stronger. These bands are too broad to be definitely assigned to a certain type (OHO or OHN) of hydrogen bonds. To correctly assign the bands, the following steps have been taken: CPMD simulations for the gas phase and the solid state (Figure 2) at 297 K (conditions for IR measurements) and 100 K (conditions for X-ray measurements). CPMD simulations in the solid state revealed the bands assigned to the OHO hydrogen bonds to be more red-shifted than those assigned to the OHN hydrogen bonds (Figure 2). This result indicates that the OHO hydrogen bonds are stronger than the OHN bonds. This fact is also supported by the completed X-ray measurements (Table 1)—the OHO hydrogen bond lengths are equal to 2.425 Å and 2.526 Å for the **4NFA-P** and **3NFA-P** complexes, respectively, which are far shorter than the OHN hydrogen bond lengths (d(ON) = 2.699 Å and 2.659 Å for **4NFA-P** and **3NFA-P**, respectively). It is worth noting that the CPMD calculations for the solid state at 297 K and 100 K show similar positions of the bands, except for the fact that the bands at 100 K are narrower than those at 297 K in view of the weaker dynamics of hydrogen at low temperature (Figure 2 and Figure A1). The spectral properties change significantly for the gas phase (Figure A1). The ν(NH) bands for the OHN hydrogen bonds are red-shifted compared to the ν(OH) band for the OHO hydrogen bonds. The ν(OH) band in the spectrum of the **4NFA-P** complex is located at 3000 cm^−1^ (Figure A2) proving the OHO hydrogen bond to be moderately strong in the gas phase. This phenomenon comes as a consequence of the strong steric repulsion between the carboxyl groups which causes the non-planar configuration of the CCO⋅⋅⋅H⋅⋅⋅OCC moiety in the **4NFA-P** complex. The ν(OH) band in the spectrum of the **3NFA-P** complex in the gas phase (Figure A2) undergoes more significant changes. The position of this band (3600 cm^−1^, Figure A2) confirms the absence of the intermolecular OHO hydrogen bond in the **3NFA-P** complex. Therefore, the substitution of the nitro group in the ortho-position of **3NFA** brings about an extremely strong repulsion between the carboxyl groups, which leads to the disruption of the intramolecular hydrogen bond and the formation of either a free hydroxyl group in the gas phase or the intermolecular OHO hydrogen bond in the solid state (see the structural analysis of the steric effect in Section 2.2). The spectral analysis presented above indicates that the **4NFA-P** complex features the formation of a short strong hydrogen bond. Similar spectral peculiarities were observed for the complex of 2,4-dinitrobenzoic acid with pyridine [43].

The comparison of the broad bands of the carboxyl groups of the studied complexes with the analogous bands of the phthalic acids studied previously [33] shows a definite and significant difference between them. The ν(OH) bands of the studied complexes are significantly red-shifted and their intensity is increased compared to that of the phthalic acids (Figure A1). This spectral result points out that the formed hydrogen bonds under the process of the phthalic acid complexation with pyridine are definitely stronger than those formed by the phthalic acids themselves in the solid state. This spectral picture is consistent with X-ray data (cf. X-ray data of the present work with the data from the paper [27]). When it comes to Raman spectra, they are less informative because of a rather weak intensity of the ν(OH) band of hydrogen bond (Figure 2).

**Figure 3:**
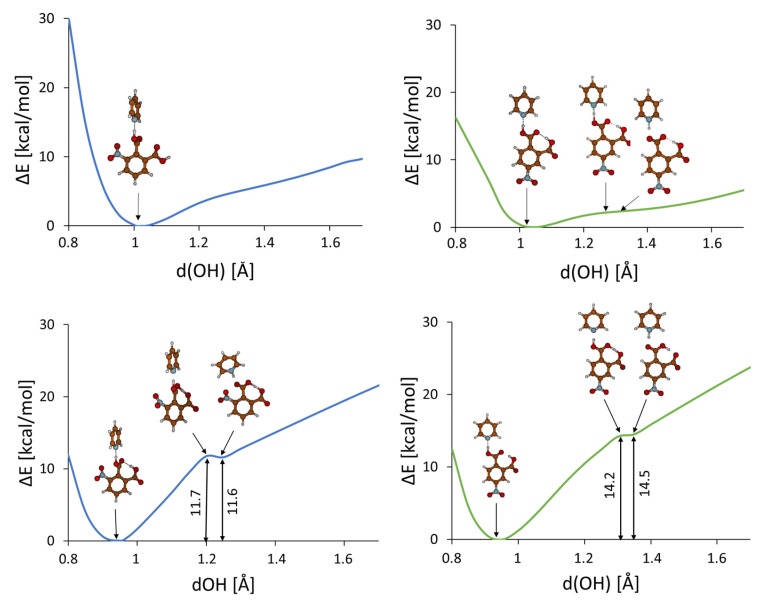
Calculated (B3LYP-D3/6-311+G(d,p)) potential energy curves for a gradual elongation of one proton within the intermolecular (upper panel) and intramolecular hydrogen bonds (bottom panel) in **3NFA-P** (left side, blue line) and **4NFA-P** (right side, green line) complexes of nitro-phthalic acids with pyridine.

Quantum-mechanical calculations were conducted to reveal the properties of transition from one isomeric state (the tautomeric state in that number) to another one in the studied complexes. DFT calculations for the proton transfer in the OH^…^N intermolecular and OH^…^O intramolecular hydrogen bonds (Figure 3) for the optimized complexes showed that the proton transfer in the OH^...^N intermolecular hydrogen bond (from carbonyl group to pyridine) is possible for both complexes. The energy difference between two tautomers (OH⋅⋅⋅N and O^−^⋅⋅⋅H^+^N) is not large and equals 9 and 10 kcal/mol for the **4 NFA-P** and **3NFA-P** complexes, respectively (Figure 3). Besides, the comparison of the potentials for the proton transfer in the intermolecular and intramolecular hydrogen bonds states that the proton transfer in the intermolecular hydrogen bond is slightly more advantageous compared to the intramolecular hydrogen bond (ΔE = 3 kcal/mol, Figure 3). The accomplished DFT calculations show that the proton transfer for the gas phase in the OHO intramolecular hydrogen bond causes a spontaneous proton transfer in the OHN intermolecular hydrogen bond (the barrier height equals 11.7 and 14.2 kcal/mol for the **3NFA-P** and **4NFA-P** complexes, Figure 3).

**Figure 4:**
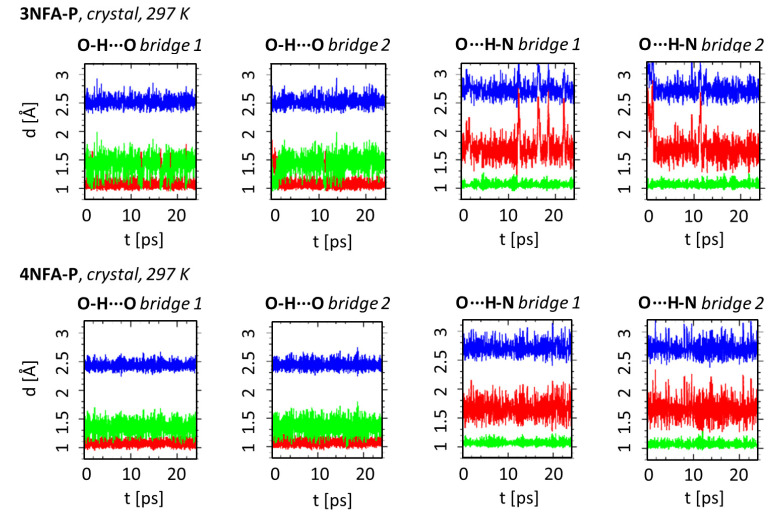
Time-evolution of the OHO and OHN hydrogen bonds metric parameters—results of CPMD solid state (T = 297 K) simulations of complexes **3NFA-P** and **4NFA-P**. The colors used in the graphs denote respectively: red–donor-proton distance, green–proton-acceptor distance, blue–donor-acceptor distance. This figure shows the dynamics of two hydrogen bridges denoted as bridge 1 and bridge 2 because two complexes are observed in crystal cell.

**Figure A3:**
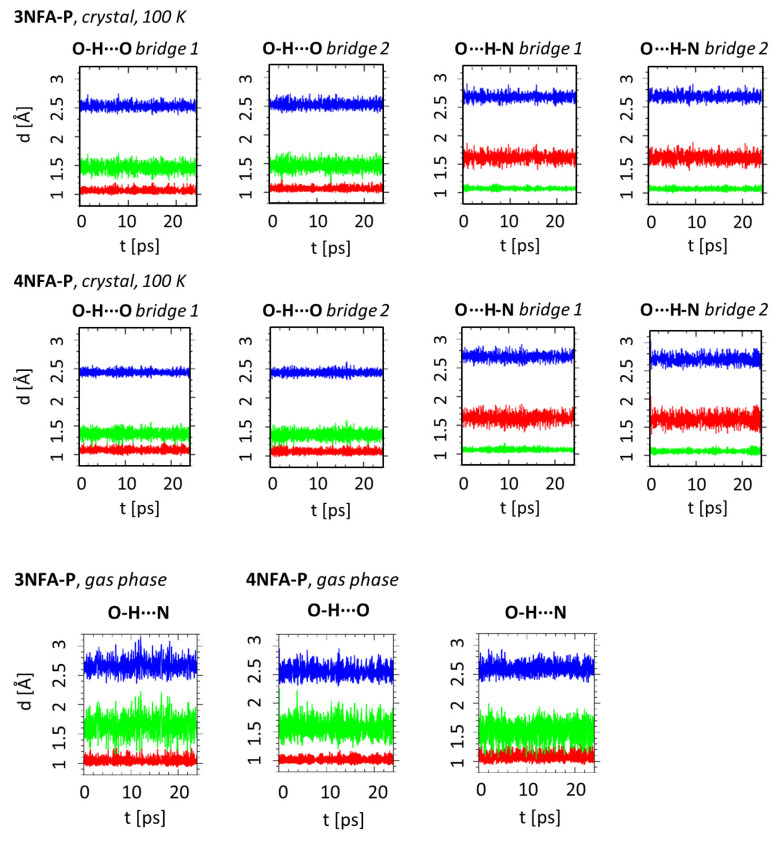
Time-evolution of metric parameters of the OHO and OHN hydrogen bonds–results of CPMD simulations in gas phase and the solid state (100 K) of the **3NFA-P** and **4NFA-P** complexes. The colors used in the graphs denote respectively: red–donor-proton distance, green–proton-acceptor distance, blue–donor-acceptor distance.

In order to study the dynamics of hydrogen bond in the obtained complexes, CPMD simulations were carried out in the gas phase and in the solid state. Figure 4 and Figure A3 show the time-evolution of the OHO and OHN hydrogen bonds metric parameters for the solid state (T = 297 and 100 K) and the gas phase. CPMD simulations were accomplished based on the obtained X-ray data for the solid state and optimized structures using B3LYP-D3/6-311+G(d,p) for the gas phase. Details of these calculations are in Section 3.4.

The time-evolution of the OH, NH and ON distances states the substantial proton dynamics in both hydrogen bonds as well as remarkable dynamics of the intermolecular OHN hydrogen bond (Figure 4 and Figure A3). This trend is observed for both complexes. Importantly, similar time evolutions are observed for the gas phase and the solid state at 297 K (Figure 4 and Figure A3). However, at temperature 100 K the dynamics of all mentioned bonds exposes a visible decrease (Figure A3), which is consistent with the reduction in the amplitude vibrations at lower temperatures. It is necessary to mention the differences in the behavior of the hydrogen bonds’ dynamics. The dynamics of the OHN intermolecular hydrogen bond are much larger than that of the OHO intramolecular hydrogen bond. The evidence is the larger amplitude of changes in the ON interatomic distance compared to the amplitude changes of the OO interatomic distance (Figure 4). The comparison of the time-evolution of the NH and ON interatomic distances for the OHN intermolecular hydrogen bond shows that significant proton dynamics in this bond are strongly conjugated with the dynamics of the bond itself. However, there is no such effect for the OHO intramolecular hydrogen bond featuring significant proton dynamics (large amplitude of the OH length changes) with moderate OHO bond dynamics (small amplitude of the ON interatomic distance changes). Such an effect can be explained by considering the following. Firstly, the intramolecular hydrogen bond is more rigid than the intermolecular one due to the covalently bonded OCCCO chain; secondly, the OHO intramolecular hydrogen bond (d(OO) = 2.425(4) Å, Table 1) is much stronger than the OHN intermolecular hydrogen bond (d(ON) = 2.699(5) Å, Table 1). This statement is based on the classification of the hydrogen bond strength presented in Refs. [44,45]. It is well known that the proton dynamics in short strong hydrogen bonds are much larger than in weak hydrogen bonds due to the much flatter potential energy surface in the former ones [46].

### 2.2. Steric Analysis Based on X-ray Results of Studied Complexes

**Figure 5:**
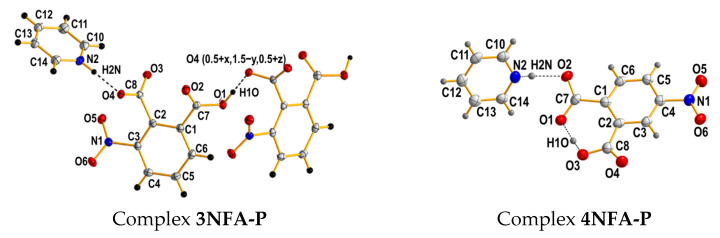
Molecular structures of the **3NFA**-**P** and **4NFA**-**P** complexes. Hydrogen bonds and bonds extending the structure are denoted with dashed lines. Displacement ellipsoids are plotted at 50% probability level.

The analysis of the measured structural data discloses a significant difference in the conformational states of the **3NFA-P** and **4NFA-P** complexes. The conformational state of the **3NFA-P** complex in the solid state is conditioned by two intermolecular hydrogen bonds and strong intramolecular steric repulsion between the carboxyl and nitro groups. This steric repulsion is strong enough to cause the turn of three groups and the formation of the non-planar structure of the **3NFA** molecule in the **3NFA-P** complex. Moreover, the network of intermolecular hydrogen bonds stabilizes the non-planar state of the **3NFA** molecule (Figure 5). Notably, the strong interaction between pyridine and the **3NFA** molecule leads to the proton transfer from the carboxyl group to the nitrogen of pyridine and the formation of the COO^−^⋅⋅⋅H-^+^N intermolecular hydrogen bond. The carboxylate (COO^−^) group forms a strong intermolecular bond (d(O⋅⋅⋅HN) = 2.526 Å, Table 1). One should stress that the carboxyl groups of the **3NFA** molecule in the studied complexes do not form the dimers characteristic of benzoic acid complexes [47,48].

When it comes to the **4NFA-P** complex, the nitro group of the **4NFA** molecule in position 4 fails to evoke the steric repulsion on the carboxyl groups; therefore, it does not hinder the formation of a short strong intramolecular OHO hydrogen bond between the carboxyl groups. Both **4NFA-P** and **3NFA-P** complexes feature the formation of the O^…^H-N intermolecular hydrogen bond with the proton transferred to pyridine. The **4NFA** molecule remains planar in the **4NFA-P** complex. To sum up, the difference between the conformational states of the studied complexes is conditioned by the steric repulsion (or its lack in the **4NFA-P** complex) of the nitro group.

## 3. Materials and Methods

### 3.1. Compounds and Solvents

The studied compounds and solvents were purchased from Sigma-Aldrich company and used without further purification. Complexes were obtained by slow evaporation from the pyridine solution.

### 3.2. Single Crystal X-ray Structure Determination of Complexes

The crystallographic measurements for the **3NFA-P** and **4NFA-P** complexes were collected with Κ-geometry diffractometer Xcalibur Gemini (Rigaku Oxford Diffraction Ltd., Oxfordshire, UK) with graphite monochromatized Cu-Kα radiation (λ = 1.5418 Å) at 100(2) K, using an Oxford Cryosystems cooler. Data collection, cell refinement, data reduction and analysis were carried out with CrysAlisPro [49]. Analytical absorption correction was applied to data with the use of CrysAlisPro. The crystal structures were solved using SHELXS [50] and refined on F^2^ by a full-matrix least squares technique with SHELXL-2015 [51] with anisotropic thermal parameters for all the ordered non-H atoms. In the final refinement cycles, H atoms were repositioned in their calculated positions and treated as riding atoms, with C-H = 0.95 Å, and with U_iso_(H) = 1.2U_eq_ (C), H atoms for N-H and O-H were found from diff. maps. All figures were prepared using the DIAMOND program [52]. CCDC 2031212 and 2031214 contain the supplementary crystallographic data for this work (Table A1). These data can be obtained free of charge via www.ccdc.cam.ac.uk/data_request/cif, by emailing data_request@ccdc.cam.ac.uk, or by contacting the Cambridge Crystallographic Data Centre, 12 Union Road, Cambridge CB2 1EZ, UK; fax: + 44(0)1223-336033.

### 3.3. Raman and Infrared Measurements

The FIR and MIR measurements were performed using a Bruker Vertex 70v spectrophotometer (Bruker Optik, Ettlingen, Germany) with a resolution of 2 cm^−1^ and with 64 scans. The FIR spectra were collected for the samples suspended in Apiezon N grease and placed on a polyethylene disc. The MIR spectra were collected in KBr pellets. The Raman spectra were obtained using FT-Nicolet Magma 860 spectrophotometer ((ThermoFisher Scientific, Madison, WI, USA,)) with a resolution of 4 cm^−1^ and with 512 scans. The In:Ga:Ar laser line at 1064 nm was employed for the Raman excitation measurements. All spectra were recorded at room temperature.

### 3.4. Car-Parrinello Molecular Dynamics in the Gas and Crystalline Phases

Car–Parrinello molecular dynamics simulations were performed using the CPMD program version 4.3 [53]. The molecular dynamics of the studied complexes were investigated in vacuo and in the crystalline phase. The models for the isolated molecule simulations were prepared on the basis of structures obtained as a result of density functional theory (DFT) [54,55]. The crystalline phase models were prepared using X-ray data (CCDC deposition number: 2031212 for the **3NFA-P** complex and 2031214 for the **4NFA-P** complex). The models for simulations in vacuo were placed into cubic cells with *a* = 12 Å for complex **3NFA-P** and *a* = 12.5 Å for complex **4NFA-P**. Initial metric parameters for the crystalline phase simulations were taken from the experimental measurements, with the following unit cell dimensions: for complex **3NFA-P** a = 7.5861(4) Å, b = 15.0232(4) Å, c = 11.3482(5) Å and β = 104.863(5)°, respectively, and with Z = 4; for complex **4NFA-P** a = 8.2455(3) Å, b = 8.5378(3) Å, c = 8.5726(3) Å and α = 87.866(3)°, β = 84.095(2)° and γ = 88.541(3)° and with Z = 2. Crystalline phase simulations were performed with periodic boundary conditions (PBCs) and with real-space electrostatic summations for the eight nearest neighbors in each direction (TESR = 8). Gas-phase conditions were achieved by removing neighborhood interactions using the Hockney scheme [56]. Concerning the gas phase and the solid state simulations, the computational setup was as follows. The exchange-correlation functional by Perdew–Burke–Ernzerhof (PBE) [57] coupled with plane-wave basis set and Troullier–Martins [58] pseudopotentials were applied during the CPMD runs. A kinetic energy cut-off for the plane-wave basis set was 100 Ry for all computed systems in both phases. The fictitious electron mass was set to 400 a.u. The time-step was set to 3 a.u. and the temperature was 297 or 100 K, controlled by the Nosé–Hoover thermostat chain [59,60]. The empirical van der Waals corrections by Grimme [61] were added during the simulations to reproduce weak interactions present in the studied systems. The investigated complexes were initially equilibrated, and then the data (production run of the CPMD) were collected for ca. 25 ps. The spectroscopic features were obtained by application of the Fourier transform of atomic velocity into vibrational signatures. The visualization and post-processing of the data were performed using the VMD 1.9.3., [62] Mercury [63] and Gnuplot [64] programs as well as home-made scripts (for Fourier transform autocorrelation function of atomic velocity).

### 3.5. DFT Calculations

The calculations were performed with the Gaussian 16 Rev. C01 program [65]. A triple zeta split-valence basis set with diffusion and polarization functions denoted as 6-311+G(d,p) [66] was applied. Static models were developed on the basis of density functional theory using the three parameters functional proposed by Becke with correlation energy according to the Lee–Yang–Parr formula, denoted as B3LYP [67,68] method. The atom pair-wise correction method for dispersion forces (DFT-D3) [61] was used. The reaction path of the bridged hydrogen was studied. The applied approach is based on stepwise elongation of the OH bond length (with 0.1 Å increment) with full optimization of the remaining structural parameters. The obtained results were visualized using the Molden 5.2 software [69].

## 4. Conclusions

This work presents results indicating the strong impact of the steric effect on the isomeric state of two complexes of nitro-derivatives of phthalic acid. The complexes of 3-nitrophthalic and 4-nitrophthalic acids with pyridine were obtained for the studies. The spectral studies of the complexes showed the formation of strong hydrogen bonds in the solid state. The performed DFT calculations revealed that the formation of the OHN intermolecular hydrogen bond provokes the appearance of the proton transfer form (O^−…^H^+^N). This result was confirmed by X-ray measurements. The CPMD results allowed us to observe that the dynamics of the proton in the OHN hydrogen bond are strongly conjugated with the vibrations of the bond itself (the change in the ON distance). X-ray measurements revealed that a short strong intramolecular hydrogen bond (SSHB) is formed between the carbonyl groups of the 4-nitrophthalic acid with the pyridine complex due to the formation of the OHN intermolecular hydrogen bond. However, this phenomenon is not observed for the complex of 3-nitrophthalic acid with pyridine due to the strong steric repulsion between the nitro group and the carboxyl groups. Such steric repulsion brings about the turn of carboxyl groups with regard to the phenyl ring and the formation of intermolecular OHN and OHO hydrogen bonds.

## Figures and Tables

**Table 1:** Structural parameters (in Å) for the donor-acceptor (O^…^O) and donor-proton (OH) distances in complexes **3NFA-P** and **4NFA-P** obtained by X-ray measurements and CPMD simulations.

	State, T	Type HB	D-H	H⋅⋅⋅A	D⋅⋅⋅A	D-H⋅⋅⋅A
complex	X-ray measurements					
**3NFA-P**	solid, 100 K	inter O(1)−H(1O)⋅⋅⋅O4^i^ (i = 1/2 + x, 3/2 − y, 1/2 + z)	0.96(2)	1.57(2)	2.526(1)	176(2)
	-	inter N(2)−H(2N)⋅⋅⋅O(4)	0.93(2)	1.74(2)	2.659(1)	173(2)
**4NFA-P**	-	intra O(1)−H(1O)⋅⋅⋅O(3)	1.33(5)	1.12(5)	2.425(4)	164(5)
	-	inter N(2)−H(2N)⋅⋅⋅O(2)	0.85(5)	1.85(5)	2.699(5)	176(3)
CPMD simulations						
**3NFA-P**	gas	inter O−H⋅⋅⋅N	1.0504	1.6515	2.6725	
	solid, 297 K	inter O−H⋅⋅⋅O	1.1138	1.4208	2.5248	
		inter N−H⋅⋅⋅O	1.7278	1.0679	2.7409	
	solid, 100 K	inter O−H⋅⋅⋅O	1.0666	1.4649	2.5277	
		inter N−H⋅⋅⋅O	1.6203	1.0716	2.6823	
**4NFA-P**	gas	intra O−H⋅⋅⋅O	1.0177	1.5933	2.5680	
		inter N−H⋅⋅⋅O	1.0962	1.5244	2.6065	
	solid, 297 K	intra O−H⋅⋅⋅O	1.0882	1.3623	2.4392	
		inter N−H⋅⋅⋅O	1.6717	1.0717	2.7176	
	solid, 100 K	intra O−H⋅⋅⋅O	1.0776	1.3651	2.4352	
		inter N−H⋅⋅⋅O	1.6301	1.0737	2.6941	

## Data Availability

Not applicable.

## Appendix A

**Table A1:** Crystal data and structure refinement for 3-nitrophthalic acid with pyridine (**3NFA-P**) and 4-nitrophthalic acid with pyridine (**4NFA-P**).

Crystal Data	4NFA-P	3NFA-P
Empirical formula	C_13_H_10_N_2_O_6_	C_13_H_10_N_2_O_6_
Formula weight	290.23	290.23
Temperature	100(2) K	100(2) K
Wavelength	1.5418 Å	1.5418 Å
Crystal system	Triclinic	Monoclinic
Space group	P-1(no.2)	P2_1_/n(no.14)
Unit cell dimensions	a = 8.2455(3) Å, α = 87.866(3)° b = 8.5378(3) Å, β = 84.095(2)° c = 8.5726(3) Å, γ = 88.541(3)°	a = 7.5861(4) Å b = 15.0232(4) Å, β = 104.863(5)° c = 11.3482(5) Å
Volume	599.74(4) Å^3^	1250.05(10) Å^3^
Z	2	4
Density (calculated)	1.607 Mg/m^3^	1.542 Mg/m^3^
Absorption coefficient	1.116 mm^−1^	1.070 mm^−1^
F (000)	300	600
Crystal size	0.19 × 0.18 × 0.07 mm^3^	0.20 × 0.20 × 0.16 mm^3^
Theta range for data collection	5.185 to 68.254°	5.891 to 68.022°
Reflections collected	3931	4776
Independent reflections	2155 [R(int) = 0.0673]	2239 [R(int) = 0.0152]
Completeness to theta = 68.254°	98.2%	98.9%
Refinement method	Full-matrix least-squares on F^2^	Full-matrix least-squares on F^2^
Data/restraints/parameters	2155/0/198	2239/0/198
Goodness-of-fit on F^2^	1.033	1.019
Final R indices [I > 2sigma(I)]	R_1_ = 0.0770, wR_2_ = 0.1902	R_1_ = 0.0286, wR_2_ = 0.0737
R indices (all data)	R_1_ = 0.1440, wR_2_ = 0.2342	R_1_ = 0.0325, wR_2_ = 0.0767
Extinction coefficient	n/a	n/a
Largest diff. peak and hole	0.340 and −0.405 e. Å^−3^	0.225 and −0.256 e. Å^−3^
